# Diagnosis and Surgical Treatment of Hydatidiform Mole

**DOI:** 10.3390/diagnostics15162068

**Published:** 2025-08-18

**Authors:** Antônio Braga, Marcela Chagas, Manisha Asrani, Juliana Pereira Soares, Sue Yazaki Sun, Edward Araujo Júnior, Rosiane Mattar, Joffre Amim Junior, Jorge Rezende-Filho, Neil S. Horowitz, Ross S. Berkowitz

**Affiliations:** 1 Department of Gynecology and Obstetrics, School of Medicine, Federal University of Rio de Janeiro, Rio de Janeiro 22240-003, RJ, Brazil; joffre@me.ufrj.br (J.A.J.); rezendef@me.ufrj.br (J.R.-F.); 2 Department of Maternal and Child Health, School of Medicine, Fluminense Federal University, Niterói 24070-090, RJ, Brazil; 3 Postgraduate Program in Applied Health Sciences, University of Vassouras, Vassouras 27700-000, RJ, Brazil; marcelavchagas@gmail.com (M.C.); ju.psoares@hotmail.com (J.P.S.); 4 New England Trophoblastic Disease Center, Division of Gynecologic Oncology, Department of Obstetrics, Gynecology and Reproductive Biology, Brigham and Women’s Hospital, Harvard Medical School, Boston, MA 02115, USA; manisha_asrani@hms.harvard.edu (M.A.); nhorowitz@mgh.harvard.edu (N.S.H.); ross_berkowitz@dfci.harvard.edu (R.S.B.); 5 Department of Obstetrics, Paulista School of Medicine, Federal University of São Paulo (EPM-UNIFESP), Sao Paulo 04023-062, SP, Brazil; sueysun@gmail.com (S.Y.S.); araujojred@terra.com.br (E.A.J.); rosiane.mattar@unifesp.br (R.M.)

**Keywords:** hydatidiform mole, gestational trophoblastic disease, diagnosis, treatment, surgery, ultrasound, uterine evacuation

## Abstract

Hydatidiform mole is a trophoblastic disorder resulting from abnormal fertilization. Diagnosis is established through a combination of clinical findings, elevated serum human chorionic gonadotropin (hCG) levels, and characteristic features on transvaginal ultrasound. Timely and accurate diagnosis is essential for initiating prompt treatment and preventing medical complications. Uterine evacuation, preferably via vacuum aspiration, is the treatment of choice due to its high efficacy and safety profile. Adjunctive techniques, such as hysteroscopy and intraoperative ultrasonography, enhance the safety and effectiveness of uterine evacuation and should be available to patients, especially at specialized referral centers equipped to manage this diagnosis. In selected cases, particularly in women with fulfilled reproductive goals or those at a high risk of developing post-molar gestational trophoblastic neoplasia (GTN), total abdominal hysterectomy is appropriate. Postoperative follow-up with serial measurements of hCG is essential for monitoring remission and for the early detection of post-molar GTN, which develops in approximately 20% of cases of complete molar pregnancies and 1–4% of partial molar pregnancies. This article provides a comprehensive review of the diagnosis of hydatidiform mole and the surgical techniques employed in the treatment of this condition, emphasizing individualized care and the use of appropriate surgical strategies to treat complications associated with this trophoblastic disease.

## 1. Introduction

Gestational trophoblastic disease (GTD) is a broad term that encompasses benign and malignant manifestations arising from the abnormal proliferation of trophoblastic tissue. Hydatidiform mole (HM), or molar pregnancy, represents a benign form of GTD and serves as a precursor lesion associated with post-molar gestational trophoblastic neoplasia (GTN), which includes invasive mole, choriocarcinoma, placental-site trophoblastic tumor, and epithelioid trophoblastic tumor [1]. Trophoblastic hyperplasia, aside from being a histopathological hallmark, also aids diagnosis through its markedly elevated secretion of a characteristic tumor marker, human chorionic gonadotropin (hCG) [2]. Early diagnosis of HM is crucial to prevent medical complications, such as potential hypertension, acute respiratory distress syndrome (ARDS), and obstetric (hemorrhagic) near-miss events [3]. Close monitoring of these patients enables the timely detection of GTN, a condition with high cure rates when appropriately treated [1,2,3].

The incidence of HM varies significantly across regions of the world, influenced by socioeconomic, genetic, nutritional, and reproductive factors. Globally, the estimated incidence is approximately 1 to 2 cases per 1000 pregnancies [4]. Nonetheless, a disparity in incidence rates has been observed in low- and middle-income countries in contrast to high-income countries. In regions such as Southeast Asia, Latin America, and parts of Africa, the incidence may be 10-to-15-times higher than in North America and Western Europe, where HM occurrence ranges from 0.5 to 1 per 1000 pregnancies [5,6]. Risk factors for HM include extremes of maternal age (younger than 15 or older than 40 years), a history of previous HM, and nutritional deficiencies, particularly of vitamin A [7].

Improved access to antenatal care, particularly early ultrasound screening, has facilitated the earlier diagnosis of HM, enabling conservative treatment and better outcomes by reducing the number of cases presenting with advanced disease [8,9,10]. Unfortunately, this early detection has not led to a similar decline in the incidence of post-molar GTN [10].

**Figure 1:**
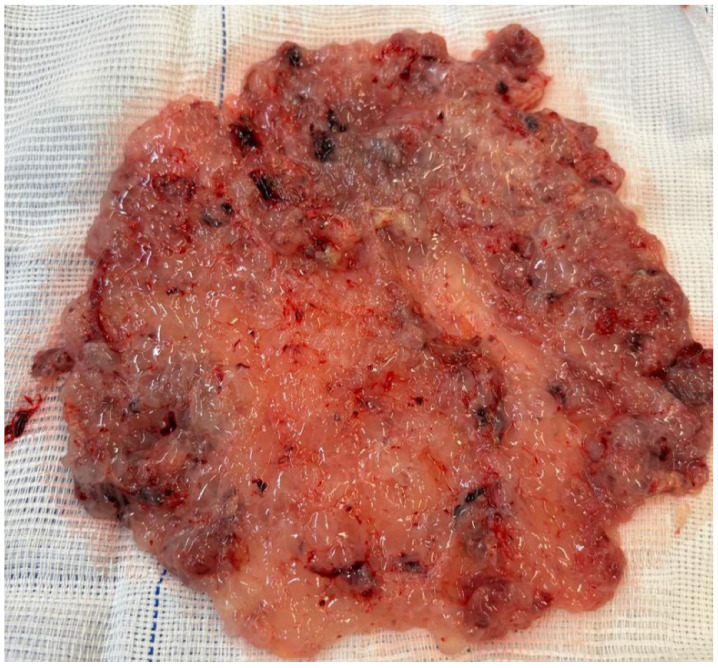
Macroscopic morphological features of a complete hydatidiform mole. The figure shows an amorphous placental mass with interspersed translucent, hydropic vesicles. Given that uterine evacuation was performed at 8 weeks of gestation, the vesicles are relatively small, consistent with morphological features typical of a complete mole at an early gestational age. Notably, there is a complete absence of embryonic and extra-embryonic features, such as the yolk sac and umbilical cord. The images refer to clinical cases managed by the authors.

**Figure 2:**
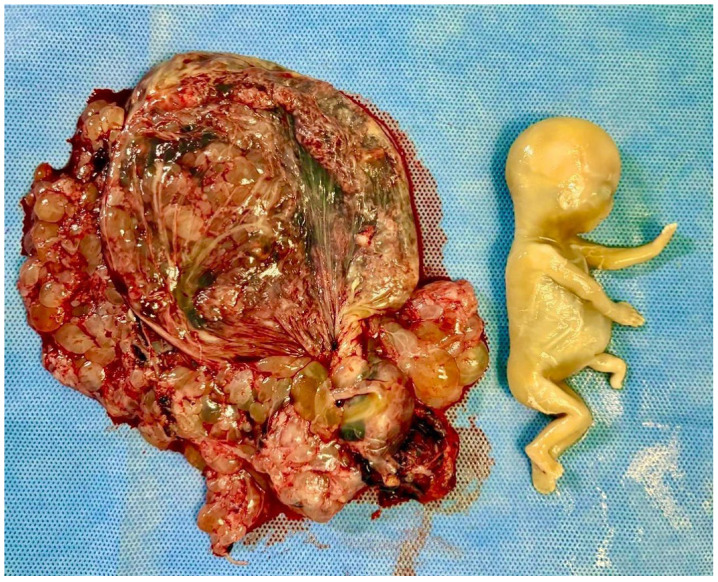
Macroscopic morphological features of a partial hydatidiform mole. This figure depicts a case of molar pregnancy terminated at 15 weeks of gestation. A fetus with an abdominal malformation with a severed umbilical cord is present. The placenta has numerous hydropic vesicles scattered across the maternal surface. The images refer to clinical cases managed by the authors.

HM originates as a consequence of abnormal fertilization and the subsequent cystic degeneration of placental tissue, which, unlike normal gestational development, predisposes it to neoplastic transformation. There are two types of HM: complete (CHM) and partial (PHM). Fertilization of an oocyte devoid of maternal genetic material by either a diploid sperm or two haploid sperms results in the formation of CHM (46,XX or 46, XY). Made up of exclusive paternal genetic material, CHM presents with trophoblastic proliferation and hydropic villi instead of a viable embryo, umbilical cord, or fetal structures (Figure 1). PHM most commonly results from the fertilization of a normal haploid oocyte by two haploid sperms (dispermy), leading to a diandric triploid conceptus (69,XXY or 69,XYY). Less commonly, PHM can arise from fertilization by a single diploid sperm due to a meiotic error. Rarely, PHM may result from the fertilization of an abnormal oocyte with retained maternal chromosomes, or present with tetraploid or other atypical ploidy patterns [2,11,12]. Unlike CHM, abnormal but non-viable fetal or placental structures may be present in these cases (Figure 2) [13].

Once diagnosed, patients should be referred to specialized GTD centers, where surgical treatment should not be delayed [14,15]. The gold standard technique used to evacuate the uterus is vacuum aspiration, which has an 80% cure rate in patients via its ability to remove trophoblastic tissue from the uterine cavity thus halting trophoblastic proliferation [1,2]. Despite complete molar uterine evacuation, 20% of patients may progress to post-molar GTN, requiring further treatment to achieve remission [16]. The rate of post-molar GTN can be as high as 40–50% in women with high-risk HMs, such as those with hCG > 100,000 IU/L, the presence of a theca lutein cyst, or a maternal age > 40 years [17].

Unlike any other malignancy, the diagnosis of post-molar GTN is primarily based on clinical characteristics and trends of hCG levels following the evacuation of a molar pregnancy. According to the criteria established by the International Federation of Gynecology and Obstetrics (FIGO), the pre-requisites for diagnosis include either a plateau of hCG levels (defined as four consecutive hCG values over three weeks that show less than a 10% variation between the highest and lowest values), rise in hCG levels (a rise of 10% or more in three consecutive weekly hCG values over at least two weeks), or a histologic diagnosis of choriocarcinoma [18].

In selected cases, prophylactic total abdominal hysterectomy (TAH) may also be indicated, which can reduce, but not eliminate, the occurrence of GTN [19]. Therefore, all patients with HM, whether treated with uterine vacuum aspiration or TAH, must undergo a rigorous post-molar follow-up to detect subsequent GTN. This follow-up involves weekly hCG monitoring, which can confirm remission, detect malignant progression, and guide further treatment [2,16,17,18]. Both early diagnosis and prompt surgical treatment are critical for minimizing the morbidity and mortality linked to this condition, which remains a significant contributor to obstetric near-miss cases and maternal deaths [3].

This review article aims to detail the strategies for diagnosis and surgical treatment of molar pregnancy, providing general practitioners and obstetrician–gynecologists, especially those less familiar with this rare condition, with clues for diagnosis and valuable surgical techniques for the management of this first-trimester obstetric emergency.

## 2. Diagnosis of Hydatidiform Mole

A combination of clinical, laboratory, and imaging characteristics aids in establishing a clinical suspicion for HM; however, a definitive diagnosis is made through histopathological findings [16,17,18].

### 2.1. Clinical Presentation

HM often presents with characteristic features, although these are less frequently seen due to earlier diagnosis via routine ultrasonography. Approximately 50% of patients experience irregular, heavy first-trimester bleeding [17]. Additional features, more commonly associated with CHM, include uterine size larger than expected for gestational age, hyperemesis gravidarum, absent fetal cardiac activity, and grape-like vesicle elimination out of the vagina (rare, but pathognomonic) [2]. HM may also present with early-onset preeclampsia (prior to 20 weeks), signs of hyperthyroidism from hCG-mediated thyroid-stimulating hormone receptor activation, or ARDS from trophoblastic embolization [2,17].

### 2.2. Laboratory Tests

Quantitative serum hCG levels are utilized to establish diagnosis, with elevated levels exceeding 100,000 IU/L, suggestive of HM. However, normal values do not exclude diagnosis, particularly in PHM or early-gestational cases [20].

In rare cases of HM, particularly CHM, levels of hCG may reach extremely high concentrations with paradoxical, false-negative readings on hCG laboratory testing due to a phenomenon known as the Hook effect or the prozone phenomenon [21,22]. This is caused by the limitation of the radiolabeled immune sandwich assay, where an excess of hCG saturates both the capture and detection antibodies, preventing antibody–antigen sandwich formation essential for a positive reading [21,22]. As a result, the assay underestimates or fails to detect true hCG levels. Clinicians should suspect the Hook effect when clinical and imaging findings strongly suggest an HM despite negative or disproportionately low hCG levels. The presence of the Hook effect can be confirmed by diluting the serum sample, which reduces excess antigen and allows for accurate quantification [20]. Recognizing this phenomenon is crucial to avoid a delayed diagnosis and management of HM [21,22].

### 2.3. Pelvic Transvaginal Ultrasound

**Figure 3:**
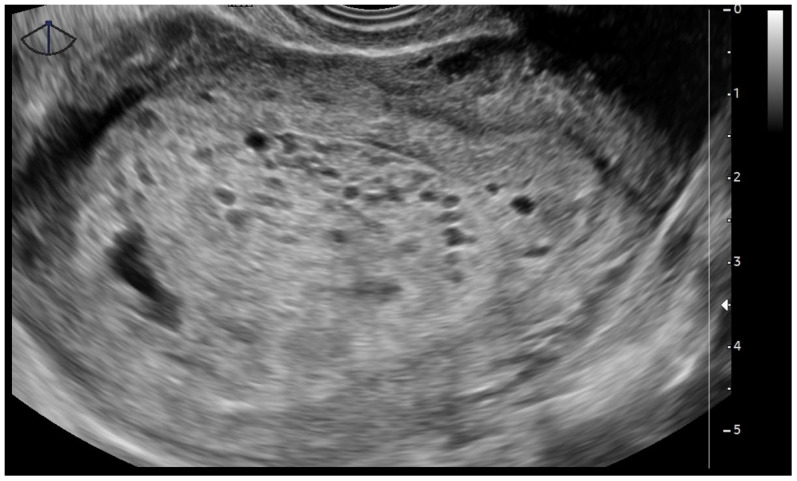
Ultrasound image of a complete hydatidiform mole. The endometrial cavity is filled with an anechoic mass characterized by multiple cystic spaces, corresponding to hydropic vesicles. There is no evidence of an embryo or associated embryonic membranes. The uterus is enlarged for its gestational age, measuring up to 12 cm in its greatest dimension, consistent with an 8-week pregnancy. The images refer to clinical cases managed by the authors.

Pelvic transvaginal ultrasonography plays a central role in diagnosis and remains the primary imaging modality. The classic ultrasonographic appearance of a CHM consists of a heterogeneous intrauterine mass with numerous anechoic cystic spaces, often described as a “snowstorm” or “cluster of grapes” pattern, which represents swollen, hydropic, chorionic villi (Figure 3) [1,2]. This pattern typically appears in the absence of a gestational sac or embryo. In some cases, the uterus may appear larger than expected for gestational age, and bilateral theca lutein cysts may be visualized in the ovaries, due to the overstimulation from markedly elevated levels of hCG [23].

**Figure 4:**
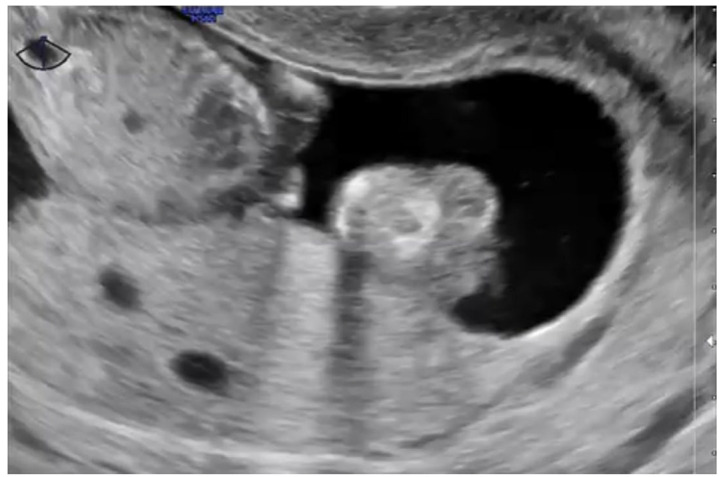
Ultrasound image of a partial hydatidiform mole. The scan reveals a non-viable fetus of an approximate gestational age of 15 weeks. The placental region shows two large anechoic regions consistent with cystic changes. The images refer to clinical cases managed by the authors.

In contrast, PHM frequently manifests with more subtle findings on ultrasound. A gestational sac with a growth-restricted fetus with hydropic areas and gross structural malformations, especially in the second trimester, is seen [24]. The placenta on visualization exhibits thickening with a snowstorm pattern of multiple echoes, reflecting hydropic changes (Figure 4) [24]. While these findings are unique to the condition, they are more common in the late-first or early-second trimester [24].

This leads to challenges with being overly reliant on ultrasound findings in women presenting at very early gestational ages due to the absence or underdevelopment of the typical morphologic features. Early CHM may not yet display the characteristic “snowstorm” or multi-cystic appearance. Instead, imaging may reveal a homogeneous echogenic intrauterine mass without a distinct gestational sac or embryo. Occasionally, a small anechoic area may be visualized, mimicking a gestational sac with the absence of a yolk sac or embryonic structures. These non-specific characteristics can closely resemble anembryonic gestation or early failed pregnancy, leading to a wrong diagnosis if not investigated further [24,25,26,27].

Similarly, in early PHM, sonographic features can closely resemble those of a missed abortion with the presence of a gestational sac and a growth-restricted or malformed fetus devoid of cardiac activity [25]. Subtle cystic spaces in the placenta are often noted but are not prominent enough to raise immediate suspicion for molar pregnancy. As a result, the misdiagnosis is recognized retrospectively on histopathological evaluation after uterine evacuation. Notably, the diagnostic accuracy of ultrasound in early PHM is considerably lower compared to CHM, necessitating clinical vigilance and follow-up with serial beta-hCG measurements [26].

In the context of multiple gestations, it is essential to distinguish a CHM coexisting with a normal fetus from other conditions, such as a vanishing twin, PHM, or placental mesenchymal dysplasia. This rare scenario, commonly referred to as a twin pregnancy with a complete mole and coexisting live fetus (CHMCF), presents significant diagnostic and clinical challenges due to overlapping imaging features and potential for severe maternal complications. Ultrasonographic evaluation is the first-line imaging modality. Typical findings include the presence of a viable fetus with a normal-appearing placenta, alongside a distinct abnormal intrauterine mass characterized by a heterogeneous echogenic pattern interspersed with multiple anechoic, cystic spaces, the classic “snowstorm” or “bunch of grapes” appearance of a CHM. This mass is usually located apart from the normally developing gestational sac and may be accompanied by theca lutein cysts in the ovaries due to markedly elevated β-hCG levels. Color Doppler may show increased vascularity within the molar component, which helps differentiate it from avascular remnants of a vanishing twin. When sonographic features are inconclusive, especially in early gestation or in cases with atypical presentations, magnetic resonance imaging (MRI) can provide additional anatomical detail and tissue characterization. On MRI, the molar component typically appears as a heterogeneous intrauterine mass with a high signal intensity on T2-weighted images and interspersed cystic areas, reflecting edematous, hydropic villi. T1-weighted sequences may show intermixed hemorrhagic foci, while contrast-enhanced studies, when used, can demonstrate diffuse enhancement in areas of trophoblastic proliferation. Importantly, MRI is valuable in assessing myometrial invasion and excluding GTN, particularly when clinical suspicion is high. When imaging findings remain equivocal, histopathological confirmation is critical. Ancillary techniques, such as immunohistochemistry for p57^kip2^ (which is absent in CHM due to its androgenetic origin), and ploidy analysis via flow cytometry or molecular karyotyping, can help differentiate a CHM from a partial mole or other placental abnormalities. A precise diagnosis is essential not only for appropriate clinical management but also for prognostic counseling, as CHMCF pregnancies carry increased risks of severe preeclampsia, hyperthyroidism, hemorrhage, and persistent gestational trophoblastic disease. Thus, early and accurate differentiation using a combination of imaging and molecular tools is critical in optimizing outcomes for both mother and fetus. [25]. Follow-up of patients after a twin molar pregnancy should include continued hormonal surveillance with weekly hCG measurements until normalization. If the criteria for GTN are met, appropriate chemotherapy should be initiated.

Ultrasonography plays a pivotal role in the initial evaluation of suspected HM, providing critical information that can guide diagnosis and management. However, several conditions may mimic the sonographic features of HM, necessitating a thorough understanding of the differential diagnoses, such as incomplete abortion, missed abortion, uterine fibroids, and ectopic pregnancies. An incomplete abortion mimics CHM via the appearance of a heterogeneous intrauterine mass with irregular echoes, but can be critically distinguished by the absence of cystic spaces and the presence of retained fetal tissue or irregular endometrial echoes. Similarly, hydropic changes in a missed abortion simulate CHM with the presence of edematous chorionic villi, but when coupled with low hCG levels, and minimal or absent trophoblastic proliferation on histopathological evaluation, hydropic changes aid in differentiation. Uterine fibroids, especially when undergoing cystic or hydropic degeneration, may appear as echogenic masses with cystic areas, potentially mimicking a molar pregnancy. Their well-circumscribed margins and typical myometrial location usually assist in differentiating them. Likewise, a negative hCG in these cases will be pivotal. Although most ectopic pregnancies are diagnosed based on the absence of an intrauterine gestational sac in the presence of a positive hCG, some complex adnexal masses can be mistaken for HM. Careful evaluation of uterine contents and adnexal regions is essential.

### 2.4. Histopathology

The histopathological examination of tissue obtained from uterine evacuation is the gold standard for establishing a definitive diagnosis of HM as well as enabling the distinction between the types of HM [15]. In CHM, the chorionic villi are uniformly enlarged and edematous, with central cistern formation and an absence of fetal vessels. The trophoblastic proliferation is diffuse, circumferential, and frequently exhibits nuclear atypia [28]. PHM consists of a mixture of normal and hydropic villi with irregular contours and focal trophoblastic hyperplasia, typically less pronounced and without significant atypia. Fetal vessels may be present, and embryonic or fetal structures are often identified, albeit abnormal and nonviable. The immunohistochemical marker p57^kip2^, which is maternally imprinted and paternally expressed, is a crucial tool in differential diagnosis: it is absent in CHM owing to its androgenetic origin and present in PHM and non-molar gestations [29]. In difficult cases, genotyping by polymerase chain reaction or fluorescent in situ hybridization may be required to confirm the diagnosis and determine the parental origin of the genome [28].

## 3. Uterine Evacuation for the Surgical Treatment of Hydatidiform Mole

Uterine evacuation should be performed in all HM patients, unless in the presence of a temporary medical contraindication that would deem this surgical approach inappropriate [2,16,17]. Factors that influence the selected surgical approach would include patient age and reproductive goals, clinical condition, uterine size, and the presence of associated complications, such as heavy vaginal bleeding or uterine perforation.

The standard technique for uterine evacuation is vacuum aspiration (manual or electric) [30]. For cases of PHM (with fetal presence and gestational age up to 13 weeks) and CHM (regardless of gestational age), vacuum aspiration is the first-line surgical treatment [31].

### 3.1. Preoperative Evaluation

Before uterine evacuation in patients with HM, a thorough clinical and laboratory evaluation is essential to ensure surgical safety, identify associated complications, and guide appropriate intraoperative and postoperative management. During the clinical assessment upon admission to the referral center, a detailed history, including identifying symptoms at presentation (such as vaginal bleeding, hyperemesis, dyspnea, abdominal pain, early preeclampsia), gestational age at diagnosis, and prior medical history, should be recorded. Physical examination should include the assessment of the general condition of the patient (pallor, dehydration, mental status), vital signs (blood pressure, heart rate, peripheral pulse quality, temperature), fundal height, and signs of hyperthyroidism or early preeclampsia [32].

Preoperative lab tests should include blood typing and crossmatching for potential transfusion, a complete blood count to assess anemia and platelet count, quantitative serum hCG with a threefold aim to support diagnosis, provide prognostic risk assessment, and establish a baseline for post-molar follow-up. Furthermore, testing for Rh is necessary as well, given the potential need to administration of anti-Rh(D) immunoglobulin [32].

In selected cases, especially in patients with clinical complications, preexisting comorbidities, or advanced gestational age (notably when uterine fundal height exceeds 16 cm), additional tests may be required, including:

▪ Coagulation profile (prothrombin time, activated partial thromboplastin time, international normalized ratio, and fibrinogen) to assess bleeding risk and coagulopathy (rare, but possible);
▪ Liver function tests (alanine aminotransferase, aspartate aminotransferase, bilirubin) in cases of early preeclampsia or systemic illness;
▪ Renal function tests (urea and creatinine), especially in patients with hypertension;
▪ Thyroid function tests (thyroid-stimulating hormone and free thyroxine) to exclude hyperthyroidism and prevent a thyroid storm during anesthesia induction;
▪ Fasting glucose for basic pre-surgical metabolic evaluation.

Pelvic transvaginal ultrasound is a critical imaging modality to confirm diagnosis as well as identify the extent of uterine and ovarian involvement, such as the presence of theca lutein cysts. When supplemented by color and spectral Doppler studies, it yields additional benefits of the evaluation of complication risk and assists in planning surgical interventions. Hemodynamic patterns, such as high-velocity, low-resistance flow characterized by a resistance index < 0.4 and a pulsatility index ≤ 1.38, aid in predicting a risk of post-molar increased GTN [33,34]. Myometrial invasion or early signs of invasive mole can also be suggested by Doppler evidence of blood flow extending beyond the endometrium into the myometrium. Furthermore, identifying areas of intense uterine vascularization can guide surgical approaches to reduce hemorrhage during evacuation.

In many centers, patients with HM undergo a pre-evacuation chest X-ray to establish a baseline evaluation of pulmonary parenchyma and detect potential lung metastases. Although computed tomography is not indicated for screening, it may be used to better characterize pulmonary lesions identified on X-ray. Chest X-ray remains recommended in the preoperative setting to detect pulmonary metastases or pulmonary embolism, and to assist in the management of ARDS, thereby helping to prevent intraoperative complications [16].

Patients in extreme clinical distress, whether due to preexisting comorbidities or previously described HM-related complications, especially uterine size exceeding 16 cm, may benefit from an electrocardiogram and echocardiography, depending on the individual’s surgical cardiac risk [32].

After completing the preoperative evaluation, all patients are required to provide informed consent, acknowledging their understanding of the procedure, its risks, and possible complications detailed in an informed consent form. These may include anesthetic accidents, intraoperative hemorrhage, uterine and pelvic organ perforation, hysterectomy, surgical site infection, and postoperative intrauterine adhesions or synechiae.

It is highly recommended for the surgeon to ensure that blood products are readily available before the surgery, especially for patients with significantly enlarged uteri or preexisting anemia, often resulting from recurrent bleeding in cases with delayed diagnosis of molar pregnancy.

### 3.2. Surgical Technique for Molar Uterine Evacuation

Uterine evacuation using vacuum aspiration, owing to its safety, effectiveness, and lower risk of complications, is the treatment of choice for HM. Concerns for complications associated with conventional uterine curettage, such as a higher risk of uterine perforation, and those with misoprostol, such as incomplete uterine evacuation, an increased risk of trophoblastic embolization, and a higher need for chemotherapy during post-molar follow-up, make these alternative evacuation options less than ideal. [31].

**Figure 5:**
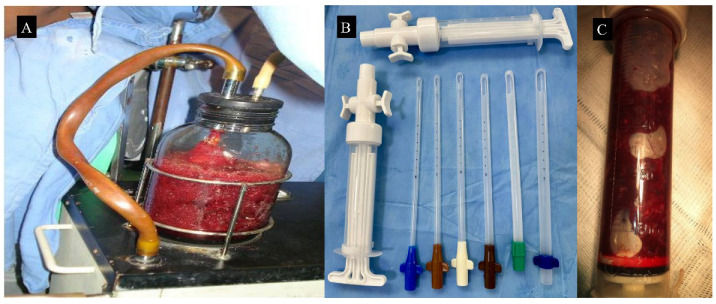
Electric and manual aspiration devices utilized for the evacuation of molar tissue. In panel (**A**), an electric vacuum aspiration device is shown. Panel (**B**) depicts a manual intrauterine aspiration syringe, along with various sizes of suction cannula. Panel (**C**) displays a syringe filled with recently aspirated molar tissue from the uterine cavity. This case involves a patient in the 9th week of pregnancy, with a pre-evacuation hCG level of 89,500 IU/L, from whom 100 mL of molar tissue was aspirated. The images refer to clinical cases managed by the authors.

Uterine aspiration can be performed using two techniques: electric (EVA) or manual vacuum aspiration (MVA) (Figure 5). Although the two techniques are equally effective in evacuating molar tissue, electric vacuum aspiration provides higher negative pressure and greater procedural control. Nonetheless, the clinical outcomes appear to be similar, and the methods differ mainly in terms of available resources and logistical requirements [30].

Table 1 summarizes the characteristics of the different surgical techniques for uterine evacuation in molar pregnancy.

A specialist oversees the anesthetic management with a preference for a combination of general intravenous and inhalation anesthesia as the preferred technique. Regional anesthesia, such as spinal or epidural, is utilized as an exception. The routine administration of prophylactic antibiotics at the time of anesthesia induction is recommended to reduce the chances of postoperative infection [30].

The procedure is performed with the patient in the gynecological lithotomy position, following meticulous vaginal antisepsis. Under anesthesia, a comprehensive and atraumatic genital examination should be performed to allow for the assessment of potential neoplastic genital lesions.

Misoprostol, a synthetic prostaglandin E1 analog, is widely used in obstetric practice to promote cervical ripening and uterine dilation; its application in the management of HM should be used cautiously. Potential complications include massive uterine hemorrhage or ARDS. Consequently, misoprostol is contraindicated in patients presenting with clinical characteristics, such as enlarged uterine size, advanced gestational age, signs of hyperthyroidism, early-onset preeclampsia or tachycardia, the presence of large theca-lutein cysts, hemodynamic instability, or clinical suspicion of invasive mole. Conversely, misoprostol may be administered exceptionally in the presence of a long and closed cervix, as a single dose in the range of 200–400 mcg administered vaginally 2 to 4 h before the surgical procedure [31]. Additional indications consist of cases of PHM with fetal biometric parameters indicative of a gestational age greater than 13 weeks.

In most cases, cervical dilatation can be effectively achieved through progressive mechanical techniques, utilizing metal dilators such as Sims or Hegar, or more safely with plastic manual vacuum aspiration cannulas. When performed by an experienced surgical team, the procedure facilitates careful and controlled cervical dilation, thereby minimizing the risk of uterine perforation and enhancing the likelihood of complete evacuation of uterine contents. The appropriate cannula size for aspiration is selected based on uterine volume, typically ranging from 7 to 9 mm in diameter. Gentle aspiration of molar tissue using circular movements of the cannula is recommended, along with the use of pelvic ultrasound guidance during the procedure for additional safety.

**Figure 6:**
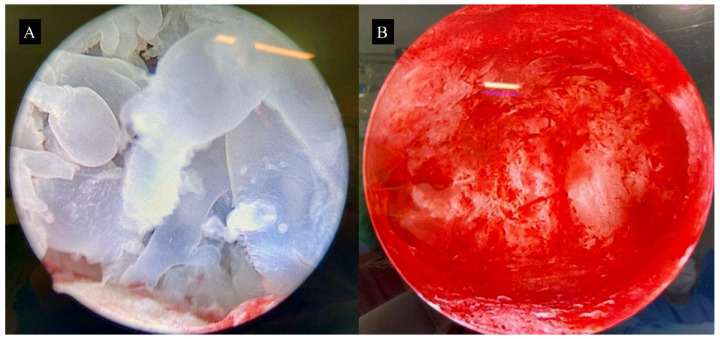
Hysteroscopic visualization before and after uterine evacuation of the hydatidiform mole. In panel (**A**), surgical video hysteroscopy reveals the presence of hydropic vesicles in the endometrial cavity. This case involves a patient at 11 weeks of gestation, with a pre-evacuation hCG level of 176,400 IU/L. The hydatidiform vesicles are abundant and enlarged. Panel (**B**) shows a follow-up hysteroscopic visualization after standard uterine evacuation, demonstrating the complete removal of all trophoblastic tissue from the endometrial cavity, indicating a successful procedure. The images refer to clinical cases managed by the authors.

In addition, surgical video hysteroscopy in carefully selected cases, performed before uterine evacuation, would enable the direct visualization of the implantation site and allow for a more targeted and effective aspiration. Following the evacuation, a hysteroscopic inspection of the uterine cavity can aid in identifying any residual molar tissue (Figure 6). Depending on the extent of the remnants, further management may include repeat aspiration or gentle removal with hysteroscopic forceps. Application of an electric current within these fragile uteri should be avoided to prevent perforation and endometrial injury that can predispose the patient to the formation of intrauterine adhesions [35]. In summary, video hysteroscopy may play a valuable role in the management of molar pregnancy, especially in selected cases. It enables direct visualization of the uterine cavity, facilitating the identification and removal of residual molar tissue after suction evacuation. When performed by experienced professionals, hysteroscopic resection may serve as a complementary approach, may reduce the need for repeat curettage, and preserve the integrity of the endometrium.

Uterine evacuation should be performed gradually and carefully to minimize the risk of uterine perforation and trophoblastic embolization. During this phase, the administration of uterotonic agents, such as intravenous oxytocin, is indicated to promote uterine contraction and reduce intraoperative hemorrhage. Curettage for revision of retained molar tissue should be carried out only in the presence of clinical suspicion by skilled surgeons, given the associated risk of uterine trauma. Routine curettage is generally discouraged in the absence of clear indications. In the event of significant uterine bleeding, a multimodal approach is advised, including the use of uterotonics, rectal misoprostol, tranexamic acid, and uterine compression.

The operating surgeon must carefully examine the aspirated material, diligently separating the blood clots from the molar tissue, to ensure that only the trophoblastic tissue is selected for histopathological analysis. The tissue specimen should be properly preserved in buffered formalin and sent to a specialized pathology laboratory [36].

Recent recommendations from the American College of Obstetricians and Gynecologists and the Society of Obstetricians and Gynaecologists of Canada question the need for anti-Rh prophylaxis in the context of uterine evacuation for first-trimester pregnancy loss [37,38]. In cases of CHM, anti-Rh prophylaxis is not required only if a diagnosis of CHM is confirmed within 72 h after an initial hemorrhage or the evacuation of the uterus, as the molar tissue lacks fetal red blood cells that can trigger maternal alloimmunization. However, in the cases of PHM, especially when gestational age exceeds 12 weeks, a more individualized approach is warranted, with a potential discussion on anti-Rh prophylaxis utility.

In uncomplicated cases of molar pregnancy, the surgical evacuation procedure generally requires a maximum of 60 min. Followed by an approximately one-hour anesthetic recovery, patients may be considered for hospital discharge within 6 to 12 h postoperatively, provided there are no clinical or surgical complications. At discharge, the patient is eligible to receive a medical certificate for leave from work for up to two weeks in uncomplicated cases. Standard postoperative care includes prescribing analgesics and contraceptives, along with a scheduled follow-up appointment within seven days of discharge. Patients should be advised to maintain contraception throughout the post-molar follow-up to ensure reliable monitoring of the serum hCG levels for the diagnosis of post-molar GTN [39,40].

## 4. Hysterectomy for the Surgical Treatment of Hydatidiform Mole

Patients who have completed childbearing or who no longer desire future fertility due to advanced maternal age are candidates for prophylactic TAH. This approach offers a preventative strategy against potential complications associated with uterine evacuation, such as uncontrollable vaginal bleeding, uterine perforation, and trophoblastic embolization. Moreover, women with advanced age constitute a high-risk population for the development of post-molar GTN and thus may experience additional benefit from prophylactic TAH [17,19].

Nevertheless, TAH does not fully eliminate the risk of post-molar GTN, which may still manifest in the form of distant metastases. Therefore, patients who undergo prophylactic TAH must undergo the same post-molar surveillance protocol as those who undergo conventional uterine evacuation [17,19]. In an effort to decrease the risk of post-molar GTN and treat possible micrometastases, some centers recommend administration of single dose of prophylactic chemotherapy at the time of hysterectomy [1].

### Surgical Technique for Hysterectomy 

Laparoscopic hysterectomy, when available and performed by experienced surgeons, is a safe and minimally invasive option associated with reduced morbidity. It is considered the preferred surgical approach for hemodynamically stable patients with molar tissue confined to the uterus. In contrast, laparotomy remains a critical option in cases of hemorrhagic emergencies, in resource-limited settings, or for patients with significantly enlarged uteri. Both surgical techniques in appropriate clinical indications are effective in managing molar pregnancy and in lowering the risk of developing GTN [41].

**Figure 7:**
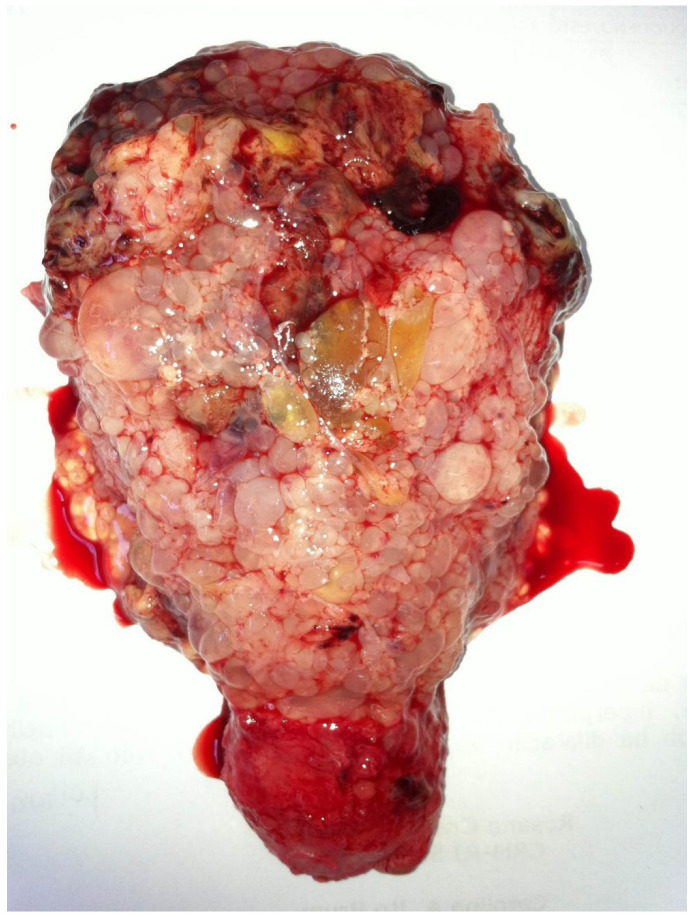
Uterus following total abdominal hysterectomy for molar pregnancy. Ultrasonographic evaluation demonstrated extensive myometrial invasion by the molar tissue, reaching the serosal surface. Considering this imaging finding, the patient’s history of completed childbearing, and advanced maternal age, hysterectomy was chosen not only to reduce the risk of uterine perforation, but also to decrease the chance of post-molar gestational trophoblastic neoplasia. The macroscopic examination shows extensive myometrial invasion by trophoblastic tissue. Despite this intervention, persistent elevation of hCG levels was observed during follow-up, necessitating chemotherapy to achieve remission. This case highlights the importance of post-molar follow-up with hormonal surveillance, even in hysterectomized patients. The images refer to clinical cases managed by the authors.

During surgery, it is crucial to handle the uterus with extreme care prior to vascular clamping, minimizing manipulation to prevent trophoblastic embolization. When performing a total hysterectomy, ovarian preservation is recommended whenever feasible. In the presence of theca lutein cysts, aspiration may be beneficial in decreasing associated morbidity. Additionally, timely salpingectomy should be considered to decrease the long-term risk of ovarian cancer [42]. All excised tissue must be sent for histopathological evaluation (Figure 7).

Postoperative management adheres to standard clinical protocols, including hemodynamic monitoring during the immediate postoperative period. Weekly serum hCG-level checks should be initiated and continued until normalization is achieved or until the diagnostic criteria for GTN, as defined by the FIGO [18], are met, at which point chemotherapy is indicated.

At the time of hospital discharge, generally around 48 h following the procedure, patients should be thoroughly counselled on potential warning signs, such as vaginal bleeding, pelvic pain, and respiratory symptoms.

## 5. Surgical Interventions for Complications of Hydatidiform Mole

Clinical and surgical complications of HM may require operative interventions to ensure clinical stabilization, prevent mutilating procedures, and preserve reproductive autonomy. These procedures are performed in the context of medical emergencies and thus demand swift, skilled execution. Their instructions and techniques are detailed below.

### 5.1. Management of Uterine Perforation

Uterine perforation can occur either concurrently during uterine evacuation or in rare cases as a spontaneous event. Uterine evacuation, while being a relatively safe procedure, can carry the risk of being complicated by perforation in 0.6% to 2.6% of cases [43,44]. The increase in uterine size, thinning of the myometrium, and pronounced softening of the uterine wall predispose CHM patients to a higher risk as compared to miscarriage. Additionally, operator inexperience, forceful instrumentation, repeated procedures, advanced maternal age, and the absence of ultrasound guidance during evacuation also contribute as risk factors.

Intraoperatively, perforation is diagnosed via a sudden loss of resistance while advancing the instrument, usually a suction cannula, or sometimes a fenestrated curette by the operating surgeon. This may be followed by unexpected and profuse bleeding, signs of peritoneal irritation, hypotension, or hemodynamic instability.

Abdominopelvic ultrasound may reveal free intraperitoneal fluid or discontinuity in the uterine wall. When ultrasound is unavailable, diagnostic paracentesis can be utilized.

Once perforation is suspected or confirmed, uterine evacuation must be halted immediately, followed by prompt hemodynamic stabilization of the patient. In cases with minimal clinical impact, a conservative approach can be adopted, involving inpatient monitoring for 24 to 48 h. Spontaneous resolution is likely when the patient remains clinically stable, has preserved bowel function, clear urine output, stable hematocrit levels, and no signs of infection or worsening [45].

In instances of extensive perforation accompanied by ongoing hemorrhage, prompt surgical intervention is warranted. In stable patients, laparoscopy is the preferred modality for assessing the abdominal cavity and uterus. This minimally invasive approach enables confirmation of the diagnosis, hemostasis for minor bleeding, suture repair of small perforations, and peritoneal lavage to remove clots or trophoblastic remnants. If intrauterine contents persist, uterine evacuation may be performed under laparoscopic visualization. This approach is optimal in centers equipped with the necessary equipment and trained personnel [46].

Laparotomy is indicated in cases of hemodynamic instability, massive hemoperitoneum, extensive or multiple uterine lesions, suspected involvement of adjacent organs (such as the bowel or bladder), or when there is an urgent need for hemorrhage control. Upon the identification of the uterine injury, repair should be undertaken using hemostatic sutures placed in two layers with absorbable material. While the abdomen remains open, uterine evacuation should be completed via the transvaginal route. This strategy is preferred for patients wishing to preserve fertility, provided the injury is localized and amenable to surgical control. However, in situations involving uncontrollable bleeding, extensive uterine trauma, or in individuals who do not desire future fertility, hysterectomy may be considered as a management option.

Regardless of whether laparoscopy or laparotomy is used, a thorough inspection of the abdominal cavity must be performed to identify potential injuries to adjacent organs. Anterior or fundal uterine perforations may commonly involve the bladder or ureters, while posterior perforations are more likely to affect bowel loops. Lateral perforations can damage pelvic vessels, fallopian tubes, or ovaries. All identified injuries should be managed appropriately, with the involvement of a multidisciplinary team comprising specialties such as general surgery, urology, vascular surgery, or colorectal surgery [47,48].

### 5.2. Management of Ruptured Theca Lutein Ovarian Cysts

Although 25–50% of patients develop theca-lutein ovarian cysts due to excessive hCG stimulation, cyst rupture is rare, occurring in approximately 1–2% of cases [49,50]. These cysts are often asymptomatic and tend to regress spontaneously as hCG levels normalize. However, large cysts may rupture, resulting in variable degrees of hemoperitoneum.

Patients should be counseled about the warning signs that include acute lower abdominal pain, often in the iliac fossa or hypogastrium, accompanied by abdominal distension, peritoneal irritation, and hemodynamic instability identified by the presence of tachycardia, hypotension, cold sweating, and near-syncope.

**Figure 8:**
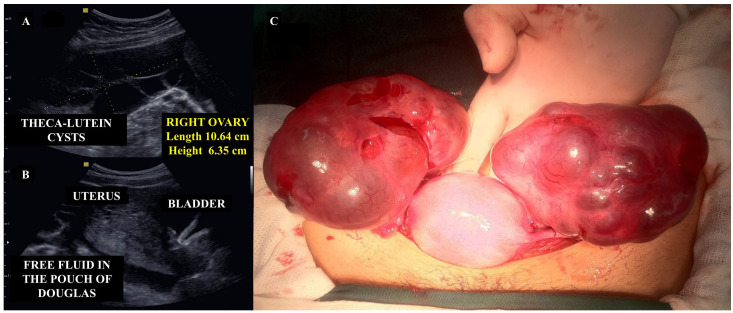
Imaging and surgical findings associated with theca lutein cysts. Panel (**A**): a pelvic ultrasound reveals a theca lutein cyst in the right ovary with a characteristic multicystic appearance. This case involves a patient at 13 weeks of gestation, with a pre-evacuation hCG level of 451,790 IU/L. Panel (**B**) shows a molar uterus accompanied by a large volume of free fluid in the pouch of Douglas, in a patient presenting with an acute hemorrhagic abdomen. There was rupture of the theca lutein cysts, resulting in hemoperitoneum. Panel (**C**) demonstrates the intraoperative findings from laparotomy consisting of theca lutein cysts with signs of rupture and active bleeding. The theca lutein cysts were aspirated, and chromic catgut 0 with a blunt needle was used for hemostatic suturing. Delicate surgical maneuvers were employed due to the extreme friability of this tissue. The images refer to clinical cases managed by the authors.

Transvaginal pelvic ultrasound can identify hemorrhagic ovarian cysts and free intraperitoneal fluid consistent with hemoperitoneum (Figure 8A,B). In some cases, pelvic CT may better assess the volume of hemoperitoneum or rule out differential diagnoses.

Management depends on the volume of hemoperitoneum, the patient’s hemodynamic stability, and future fertility goals. Expectant management is acceptable in stable patients with controlled pain, no significant peritoneal signs, small-to-moderate hemoperitoneum, and minimal hematocrit decline. In these cases, 24–48 h of hospital observation allows the monitoring of vital signs, adequate analgesia, and fluid resuscitation if needed. Serial ultrasound after discharge monitors hemoperitoneum resolution [51].

Surgery is indicated in patients with hemodynamic instability, overt hypovolemic shock, significant peritoneal irritation, large-volume hemoperitoneum (>300–500 mL), or clinical deterioration during conservative treatment. The choice between laparoscopic and open surgical approaches should be determined by the one that is most readily accessible and feasible within the clinical setting. Intraoperatively, the peritoneal cavity should be aspirated, hemostasis achieved via control of bleeding vessels, along with repair of the ovarian capsule or cyst wall (Figure 8C). Aspiration of the cyst may be necessary before suturing to facilitate repair. If these interventions are unsuccessful or if the ovary is found to be extensively damaged or necrotic, partial or total oophorectomy may be warranted [51,52].

### 5.3. Conservative Surgical Management of Uterine Hemorrhage

Uterine hemorrhage is the most common clinical complication of HM. Although most cases are effectively managed with uterine evacuation and medical therapy, persistent or recurring bleeding that fails to respond adequately and results in hemodynamic instability may necessitate surgical intervention. Hemorrhage is often attributed to retained molar tissue, for which repeat suction evacuation is indicated. The use of ultrasound or hysteroscopic guidance may enhance surgical accuracy and reduce associated risks.

Temporary hemostatic interventions, such as uterine tamponade, may also be utilized to control bleeding. While the Bakri balloon is generally too large for uteri of early-pregnancy size, adapted Foley catheters can serve as an effective alternative, providing critical time for patient stabilization or transfer to a specialized care facility.

In more severe cases, when conservative management is still desired to preserve uterine integrity, vascular ligation, typically of uterine or internal iliac arteries, may be considered as a final measure. This technique promptly reduces uterine perfusion, thereby facilitating hemostasis.

Uterine artery embolization (UAE), a minimally invasive procedure performed by interventional radiologists, represents another valuable therapeutic option for managing persistent uterine hemorrhage in HM patients [53]. Temporary uterine ischemia effectively reduces bleeding, promotes trophoblastic necrosis, and preserves fertility. This procedure involved percutaneous access via femoral artery puncture, followed by selective catheterization of the internal iliac and uterine arteries, typically on both sides. Contrast-enhanced imaging identifies active bleeding (extravasation), which is then embolized using agents, such as polyvinyl alcohol particles, gel foam, or calibrated microspheres. Successful occlusion is confirmed via post-embolization angiography. UAE is associated with immediate bleeding control in over 90% of cases, along with reduced recovery time, minimal intraoperative blood loss, and a decreased likelihood of requiring major surgical procedures, such as hysterectomy. However, the procedure carries some risks, including pelvic pain owing to post-embolization syndrome, infection at the puncture site or within the uterus, decreased ovarian reserve, particularly when collateral ovarian vessels are affected, temporary or permanent amenorrhea (mostly in women > 40), and in extreme cases, uterine necrosis [54].

### 5.4. Management of Ectopic Hydatidiform Mole

Ectopic HM is an exceedingly rare but serious condition, characterized by the implantation of molar tissue outside the uterine cavity, most commonly within the fallopian tube. Owing to its rarity and clinically similar presentation to ectopic pregnancies, the diagnosis is frequently made retrospectively following a histopathological evaluation of tissue obtained during surgical management. Early and accurate identification is crucial to mitigate complications such as hemorrhage, tubal rupture, and progression to post-molar GTN [55,56].

The clinical presentation mimics that of a non-molar ectopic pregnancy: lower abdominal pain, vaginal bleeding, amenorrhea, and elevated serum hCG, notably higher levels than in typical ectopic pregnancies but lower levels than in cases of intrauterine molar pregnancies.

In suspected ectopic HM, an ultrasound may reveal the absence of an intrauterine gestational sac and the presence of a heterogeneous adnexal mass with multiple small anechoic areas. Doppler imaging may show increased vascularity within the lesion, although these findings are not specific. Definitive diagnosis requires a histopathological confirmation of hydropic villi and exuberant trophoblastic proliferation in tubal tissue.

The treatment plan is individualized based on the location, clinical presentation, hemodynamic stability, and reproductive goals of the patient. Unlike non-molar ectopic pregnancies, where expectant or medical management may be possible, ectopic HM requires surgical treatment. Salpingectomy is the most common approach, especially in the setting of tubal rupture or active bleeding, to ensure complete molar tissue removal [57]. Methotrexate is reserved for cases where GTN is diagnosed postoperatively as per FIGO criteria [18].

### 5.5. Cesarean Section in Twin Pregnancies with Coexisting Hydatidiform Mole

Among the various forms of molar pregnancy, twin gestations comprising a coexisting HM and a viable fetus are one of the rarest presentations of HM recorded in the literature. The estimated incidence for a dizygotic twin pregnancy with a diploid, potentially viable fetus alongside an HM, most commonly CHM, is 1 in 20,000–100,000 pregnancies. Apart from its clinical rarity, this presentation presents major diagnostic and management challenges due to high maternal and fetal risks [58,59,60].

**Figure 9:**
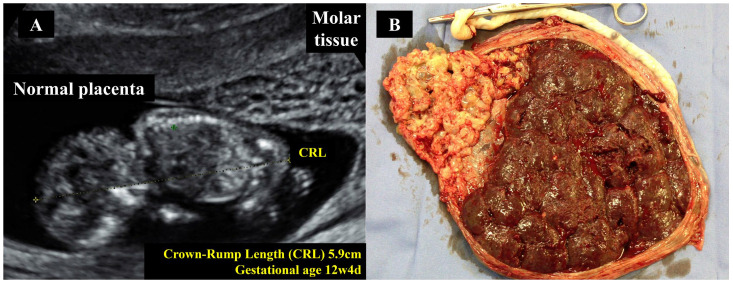
Twin pregnancy with a coexisting complete mole. In panel (**A**), obstetric ultrasonography at 17 weeks of gestation shows a fetus adjacent to a heterogeneous placenta, exhibiting both areas of normal morphology alongside multiple anechoic regions. These findings suggest a twin pregnancy with a coexisting mole. Fetal karyotyping via amniocentesis confirmed a diploid genome. With close prenatal monitoring, the pregnancy progressed to term and was delivered by cesarean section due to complete placenta previa. The infant weighed 2700 g with Apgar scores of 8 and 9 at one minute and five minutes, respectively. Panel (**B**) shows the placenta post-delivery, with a distinct molar region confined within otherwise normal placental tissue. The images refer to clinical cases managed by the authors.

On ultrasound examination, features of molar tissue, such as an echogenic mass containing multiple anechoic spaces and prominent vascularization, coexist with a viable fetus with normal placentation (Figure 9A). Fetal euploidy and the decision to continue pregnancy are made based on invasive genetic testing, such as amniocentesis, and the health status of the mother. However, 40–60% of such pregnancies are terminated due to early maternal complications, including massive hemorrhage, early-onset preeclampsia, maternal hyperthyroidism, acute respiratory distress syndrome, fetal growth restriction, preterm birth, or progression to GTN [58].

Expectant management in high-risk antenatal care with thorough monitoring via a multidisciplinary team is ideal for cases where patients are clinically stable and fetal development is satisfactory. In the event of complications, pregnancy should be terminated regardless of gestational age [59].

Due to the advantages for both the mother and the fetus in these scenarios, a cesarean section is the advised birthing approach. From the perspective of the mother’s fragile and hyper-vascular uterus, and the associated risk of massive hemorrhage during vaginal delivery along with the possibility of retention of molar tissue, subsequent infection and progression to post molar GTN cesarean section seems to be the ideal choice. Additionally, due to aberrant placentation, women also present with placenta previa and an increased volume of placental tissue, supporting the choice of opting for a cesarean section as the preferred mode of delivery.

The fetal factors that contribute to this include high rates of intrauterine growth restriction and fetal distress. Furthermore, prematurity and frequent maternal instability reinforce cesarean as the safest option [61].

For twin molar pregnancies, cesarean section for delivery must be performed in a hospital with intensive maternal and childcare, along with access to blood transfusion services. To minimize trophoblastic embolization, the molar tissue should be carefully removed during the procedure, followed by histopathological and genetic evaluations of all placental and molar tissue as good clinical practice (Figure 9B).

## 6. Conclusions

Early diagnosis of HM is critical for preventing serious clinical complications and improving patient outcomes. Timely recognition through clinical evaluation, quantitative hCG testing, and high-resolution pelvic transvaginal ultrasound allows for appropriate and prompt management, significantly reducing medical complications at presentation. Ultimately, increased awareness and accurate early detection of HM are essential components in safeguarding the reproductive health and overall well-being of affected women.

The surgical treatment of HM remains a cornerstone in the safe and effective management of this condition, whose clinical presentation can range from asymptomatic cases to severe hemorrhagic emergencies. Uterine evacuation using vacuum aspiration, either electric or manual, is the preferred method due to its effectiveness in removing trophoblastic tissue completely and its lower risk of complications compared to other techniques. In specific situations, such as uterine perforation or uncontrollable hemorrhage, additional surgical approaches may be necessary, including hysterectomy or conservative procedures, such as selective UAE. The choice of surgical technique for the treatment of HM should always consider the patient’s clinical stability, future reproductive desires, and the medical team’s experience.

Regardless of the initial treatment, strict follow-up with serial hCG measurements is essential for the early detection of post-molar GTN. Ultimately, when performed in a careful and individualized manner, surgical management of HM plays a key role in reducing morbidity and mortality and preserving the reproductive health of patients affected by this trophoblastic disease.

## Figures and Tables

**Table 1:** Characteristics of surgical techniques for uterine evacuation in molar pregnancy by electric or manual vacuum aspiration.

Characteristic	Electric Vacuum Aspiration	Manual Vacuum Aspiration
Vacuum source	Electric pump	Manual plastic syringe
Negative pressure	Controlled by electric device (±600–700 mmHg)	Manually generated (±400–500 mmHg)
Aspiration control	Continuous and adjustable	More operator-dependent
Required equipment	Electric pump, cannulas, connection tubing	Sterilizable or disposable syringe, cannula
